# Evidence of Key Tinnitus-Related Brain Regions Documented by a Unique Combination of Manganese-Enhanced MRI and Acoustic Startle Reflex Testing

**DOI:** 10.1371/journal.pone.0014260

**Published:** 2010-12-15

**Authors:** Avril Genene Holt, David Bissig, Najab Mirza, Gary Rajah, Bruce Berkowitz

**Affiliations:** 1 Department of Anatomy and Cell Biology, Wayne State University School of Medicine, Detroit, Michigan, United States of America; 2 Department of Ophthalmology, Wayne State University School of Medicine, Detroit, Michigan, United States of America; Hotchkiss Brain Institute, University of Calgary, Canada

## Abstract

Animal models continue to improve our understanding of tinnitus pathogenesis and aid in development of new treatments. However, there are no diagnostic biomarkers for tinnitus-related pathophysiology for use in awake, freely moving animals. To address this disparity, two complementary methods were combined to examine reliable tinnitus models (rats repeatedly administered salicylate or exposed to a single noise event): inhibition of acoustic startle and manganese-enhanced MRI. Salicylate-induced tinnitus resulted in wide spread supernormal manganese uptake compared to noise-induced tinnitus. Neither model demonstrated significant differences in the auditory cortex. Only in the dorsal cortex of the inferior colliculus (DCIC) did both models exhibit supernormal uptake. Therefore, abnormal membrane depolarization in the DCIC appears to be important in tinnitus-mediated activity. Our results provide the foundation for future studies correlating the severity and longevity of tinnitus with hearing loss and neuronal activity in specific brain regions and tools for evaluating treatment efficacy across paradigms.

## Introduction

Tinnitus, the perception of a ringing, buzzing, or hissing in the absence of an external stimulus due to noise, drugs, or traumatic brain injury, is a rapidly growing major health concern affecting both civilian and military populations [1], [2], [3], [4]. Mounting evidence suggests that tinnitus can be linked with increased spontaneous neuronal activity [5], [6], [7], [8] leading to the use of drugs targeted at reducing spikes of increased activity. However, such approaches have only been partially successful [5], [9], [10], [11], [12]. A better understanding of the pathophysiology of tinnitus is needed to improve future treatment efforts.

Animal models of tinnitus (e.g., noise or drug induced) produce increases in spontaneous neuronal activity, which are likely downstream consequences of changes in calcium and calcium channel-dependent neurotransmitter release. Blocking calcium channels with nimodipine has been shown to reduce percepts associated with salicylate-induced tinnitus [13], [14], [15], [16]. Despite such promising results, there is a pressing need to analytically measure calcium ion regulation in auditory-related brain regions to determine whether or not common brain regions and abnormalities exist across tinnitus models.

**Figure 1:**
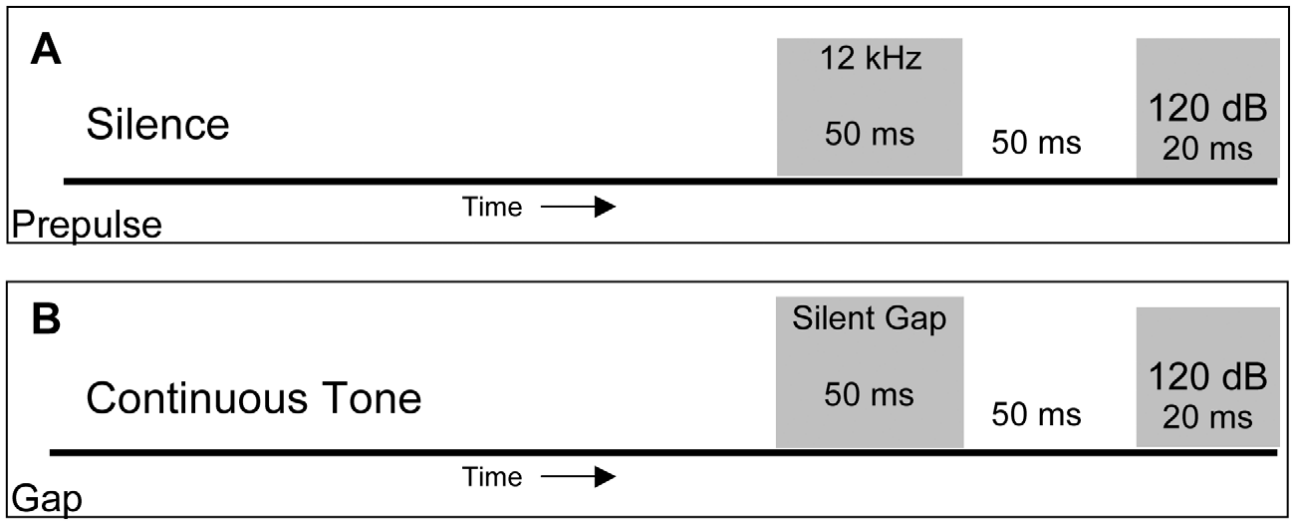
Schematic of acoustic startle reflex (ASR) under conditions of pre-pulse (pASR) or gap (gASR) inhibition. (**A**) During the pASR test animals are presented with a randomly introduced 120 dB stimulus resulting in a measurable startle response. If a 50 ms tone preceding a 50 ms silent period is introduced and detected just prior to the startle stimulus, usually a decreased startle reflex is observed. (**B**) When a startle stimulus is presented in the presence of a continuous background tone (4,8,12, 16, 20, or 24 kHz) a startle reflex is elicited. If a 50 ms silent gap in the background tone is presented just prior to the startle stimulus then the startle response is decreased. However, if an animal has tinnitus percepts and the tone matches the tinnitus in frequency and loudness then the silent gap is filled in by the animal's tinnitus and the startle response is not significantly blunted.

A recent methodological advance in the tinnitus field involves the use of gap and pre-pulse inhibition of the acoustic startle reflex (ASR) for screening of tinnitus percepts in animal models. The paradigm was designed to assess whether the subject perceives tinnitus by introducing a pre-stimulus “gap” in sound (silence) just prior to the startle stimulus. If the pre-stimulus gap is detected, there is a decreased startle reflex observed when the startle stimulus is presented (Figure 1A–B). If the gap is embedded in a tone that closely matches the animal's tinnitus in frequency and intensity, the result is a full startle reflex that is not blunted [17]. While such psychophysical tests are useful in the assessment of tinnitus percepts, they cannot spatially resolve pathological changes in specific auditory regions and so miss important mechanistic information regarding the initiation and maintenance of tinnitus.

Manganese-enhanced MRI (MEMRI) with systemically administered MnCl_2_ has been used to examine neuronal activity *in vivo* [18], [19], [20]. The paramagnetic Mn^2+^ ion (manganese) can enter active neurons through voltage-gated calcium channels, amongst others calcium channels [21], [22], [23], [24], [25]. Since manganese efflux from cells is slow [26], activity-dependent accumulation of the ion in brain regions can be measured as a decrease in tissue T_1_ at high spatial resolution hours later [27], [28], [29]. In this manner, MEMRI has been used to measure sound-evoked activity in the midbrain from awake and free moving rodents [20], [30]. To-date only one study has used MEMRI to investigate tinnitus, however, analysis was performed in excised tissue [18]. Whether manganese accumulation in auditory-related brain regions will be sensitive enough to detect differences between awake and freely moving animals with and without tinnitus or between groups with tinnitus produced by different means (noise, salicylate, blast wave, etc.) remains to be determined.

In this study, behavioral (ASR) and imaging (MEMRI) assessments were combined to identify and characterize early stages of tinnitus in two different animal models. We have identified a common brain region with increased activity regardless of the method of tinnitus induction, and brain regions in which activation is specific to the method used to induce the tinnitus.

## Results

### Salicylate and noise exposure result in temporal differences in hearing loss

Relative to that in controls (average of 30 dB across three frequencies), injection of salicylate results in significant changes in hearing thresholds (32 dB; p = 0.04) when animals were tested two hours following administration on the second day of treatment (Table 1). After 10 hours with no additional salicylate hearing thresholds returned to normal (Table 2). Therefore, daily administration of salicylate resulted in daily temporary threshold shifts averaging 1–12 dB across frequencies depending upon the time after salicylate administration. In contrast, animals that were exposed to a 10 kHz 118 dB 1/3 octave band noise generally exhibited profound deafness (Table 1) with average thresholds of 98 dB or more in the ear that was not plugged (p = 6.0 E^−6^) and elevated thresholds averaging of 51 dB across the three frequencies, tested in the plugged ear, that were not significantly different from control thresholds (p = 0.09). Two animals that were noise exposed, but were not imaged had ABR assessment at both 24 and 48 hours with animals tested at 48 hours showing no significant change in hearing thresholds from the 24 hour time point (data not shown). Therefore, in contrast to salicylate-induced changes in hearing threshold, we found no evidence for diminished noise-induced changes in this study. Therefore, changes in hearing, as measured by ABR, were temporary for salicylate-injected animals and permanent (bilaterally, although asymmetrical) for sound exposed animals (Tables 1 and 2). To determine whether these temporal differences in hearing loss would differentially affect tinnitus percepts we recorded pASR and gASR across both animal models of tinnitus and normal hearing controls.

**Table 1:** Summary of hearing thresholds following salicylate or noise treatment: Auditory brainstem responses.

Frequency	4 kHz		12 kHz		20 kHz		Average		P-value a	
Treatment	*Left*	*Right*	*Left*	*Right*	*Left*	*Right*	*Left*	*Right*		
Control b, c	31±5.74	29±3.85	29±2.49	28±2.40	30±4.77	30±3.30	30±4.33	29±3.18	-	
Salicylate b, c	30±2.50	33±3.50	32±3.95	32±2.38	31±3.11	32±2.22	31±3.01	32±2.62	0.05	
Noise I b, c	**33±6.85**	96±9.00	**65±22.81**	100↑	**62±19.25**	100↑	**53±21.80**	99±5.20	**Noise-Plugged**	**0.16**
									Noise-Unplugged	0.004
Noise II b, c	90±14.14	**30±2.12**	100↑	**58±14.14**	100↑	**51±24.04**	97±8.16	**46±18.25**	**Noise-Plugged**	**0.19**
									Noise-Unplugged	0.001

a Significance p≤0.05.

b Hearing threshold, expressed in decibels (dB).

c Error (±) is expressed as SEM.

100↑More than 100 dB.

Bold indicates the protected (plugged) ear (Noise I left ear protected Noise II had the right ear protected).

**Table 2:** Hearing thresholds at two time points following salicylate administration: Auditory brainstem responses.

Frequency	4 kHz	12 kHz	20 kHz
Treatment			
Control a,b	30±5.04	28±2.45	30±4.15
6 hrs after injection a,b	34±2.99	34±2.50	33±2.89
10 hrs after injection a,b	30±2.22	30±1.73	30±1.50

a Hearing threshold, expressed in decibels (dB).

b Error (±) is expressed as SEM.

### ASR demonstrates specific tinnitus percepts specific to the animal model

**Figure 2:**
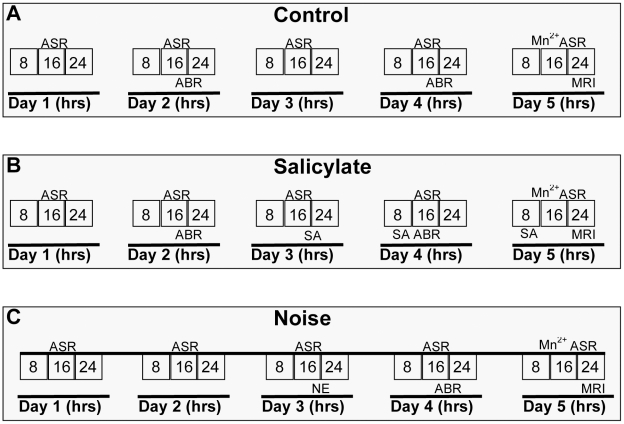
Time line showing day and approximate time of day for each procedure. Acoustic startle reflex testing (ASR) for tinnitus perception, auditory brainstem response testing (ABR) for hearing loss, tinnitus induction, manganese injection and imaging in control (**A**), salicylate (**B**) and noise (**C**) groups during a given day. 8 – first eight hrs of day, 16 – second eight hr period of day, 24 – last eight hr period of day, SA – salicylate injection, NE – noise exposure, Mn^2+^ - manganese injection, MRI – magnetic resonance imaging.

To compare behavioral correlates of salicylate and noise induced tinnitus, the acoustic startle reflex was tested and compared at three time points per group, before salicylate treatment or noise exposure (baseline 2–3 measures), after salicylate administration (after one hour on day two) or noise treatment (after 24 hours) as well as on the day of imaging seven hours after the final administration of salicylate or 55 hours following noise (Figure 2).

For ASR testing animals needed to demonstrate the ability to exhibit a robust startle reflex in response to a brief loud noise as well as the capacity to attenuate the startle reflex in the presence of a pre-pulse or a silent gap in a tone. Of the animals tested for startle reflex under baseline conditions, all of the animals exhibited both a robust sound off startle reflex as well as the ability to inhibit the reflex in approximately 70% of the 120 total trials - - ten trials recorded for each of the six different frequencies tested at two different sound levels (45 and 60 dB for control and salicylate groups, 70 and 95 dB for the noise exposed group).

**Figure 3:**
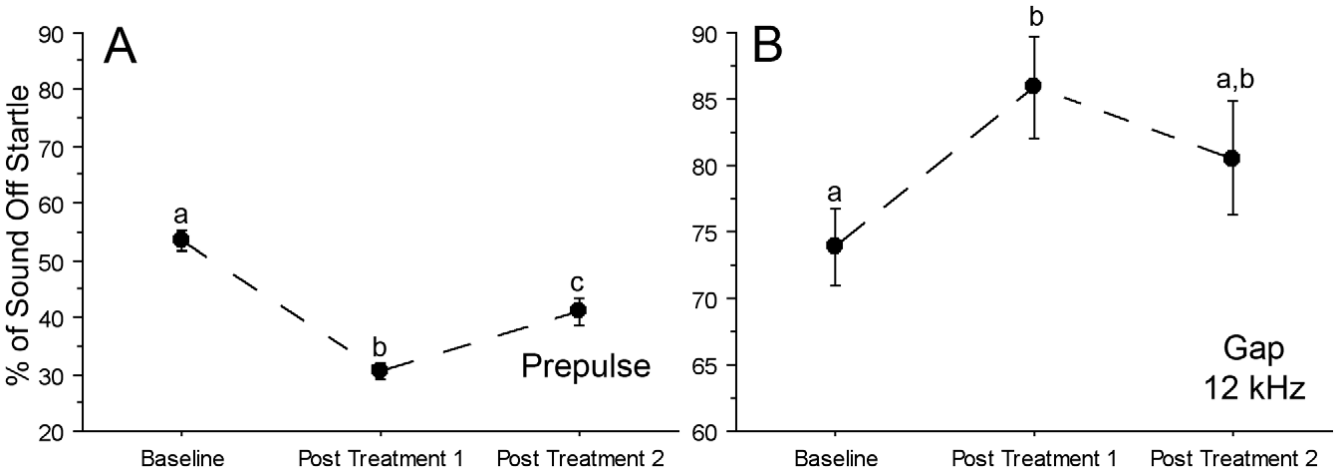
Salicylate results in a decreased ability to blunt the acoustic startle response (ASR) under conditions of gap inhibition. Performance during ASR testing was recorded before treatment (Baseline), 1 hr following the second day of salicylate administration (Post Treatment 1) and seven hrs following the third day of salicylate administration (Post Treatment 2). Relative to a full startle response (100%), untreated rats detected the gap and prepulse and could suppress the startle reflex across all frequencies (baseline in **A**). Across all frequencies significant decreases in pASR were observed relative to baseline both one hour following salicylate administration (Post Treatment 1) and seven hours after treatment (Post Treatment 2) (**A**). When compared to baseline, p = 0.0001 for both Post Treatment 1 and 2 (compare a–b and a–c). Post Treatment 1 and 2 were also significantly different from one another (b–c; p = 0.0004). At 12 kHz the % sound off startle initially changed by 12% (p = 0.05) from Baseline to Post Treatment 1, but was not significantly different from Baseline during Post Treatment 2 testing (**B**). Differing letters denote significance across time points, p≤0.05 was significant; error bars equal SEM.

Control animals showed a robust attenuation of both the pASR and the gASR regardless of the frequency that was tested (Figure 3). However, inhibition of the acoustic startle reflex was less variable at 60 dB when compared to 45 dB (data not shown) suggesting that the higher intensity pre-pulse or background tones increased ASR sensitivity when comparing performance across frequencies. Therefore results are presented for the 60 dB levels.

**Figure 4:**
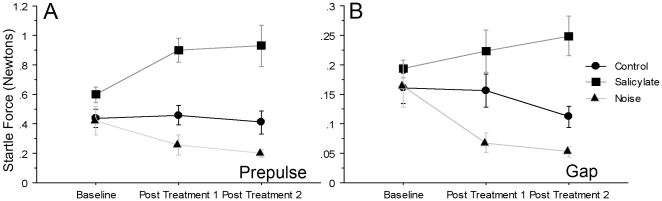
Treatment with salicylate or noise results in changes in startle force elicited by the startle stimulus. There was no significant difference in Baseline acoustic startle response (ASR) values during Prepulse (**A**) or Gap (**B**) tests when comparing control, salicylate and noise groups. Follwing salicylate treatment there were significant differences in startle force when compared to controls during prepulse testing for both Post Treatment 1 and Post Treatment 2 (p≤0.0001 in each case) as well as gap testing (Post Treatment 2 p = 0.002). For the noise treated group there no significant differences in startle force when compared to controls during prepulse testing for Post Treatment 1 or Post Treatment 2 (p = 0.093) nor gap testing for Post Treatment 1 or 2 (p = .09). p≤0.05 was significant; error bars equal SEM.

In pre-drug baseline testing, rats in the salicylate group detected either the prepulse (Figure 3A) or the gap (Figure 3B) and demonstrated the ability to suppress the startle reflex. Across frequencies, pASR testing revealed robust suppression of the startle reflex (46.5%±1.69) prior to treatment. Greater suppression was observed one hour after salicylate treatment on the second day (69.5%±1.50; p<0.0001), and seven hours after salicylate treatment on the third day (59%±2.34; p<0.0001; Figure 3A). Relative to the full startle response amplitude (100%) gASR testing at 12 kHz (Figure 3B) resulted in a robust attenuation of startle (26%±2.92). After salicylate treatment, gASR startle amplitude was significantly increased at 12 kHz (12%±3.85; p = 0.05) over baseline, but was not significantly different from baseline 7 hours post treatment (Figure 3B). In addition, salicylate treated animals exerted significantly more force in response to the startle stimulus only when compared to controls both during pASR (Figure 4A) testing (0.90±0.08; p<0.0001 1 hr after injection; 0.93±0.14; p<0.0001 seven hrs after injection) and gASR (Figure 4B) testing (0.22±0.04; p = 0.02 1 hr after injection; 0.25±0.03; p≤0.05 seven hrs after injection). Thus, gASR testing demonstrated cyclical salicylate-induced tinnitus as shown by ABR (Table 2), while pASR testing demonstrated a more sustained hyperacusis (Figure 3A) with both tests reflecting increased force following treatment when comparing force exerted in response to startle stimulation only (Figure 4).

**Figure 5:**
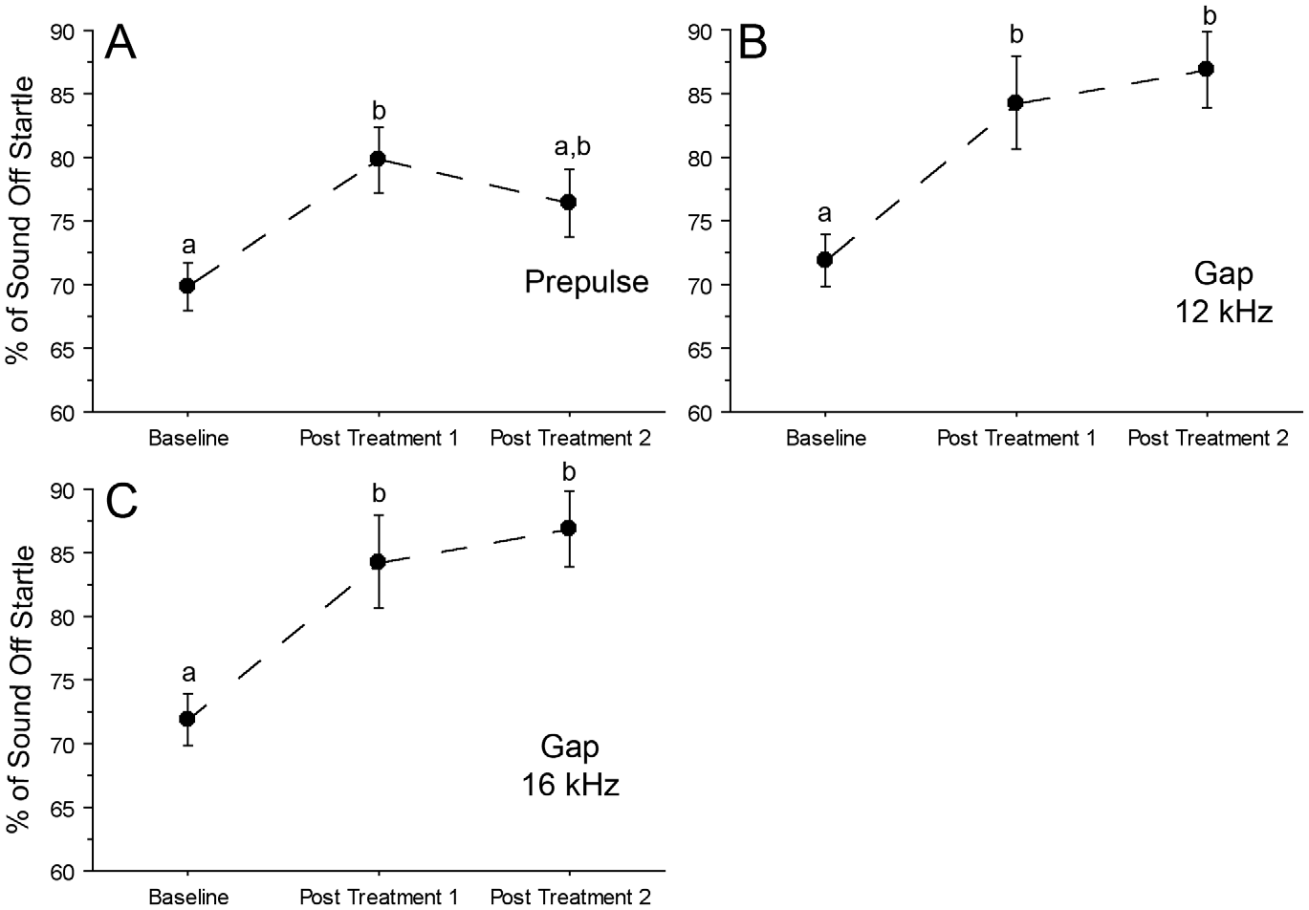
Hearing loss (pre-pulse) and tinnitus (Gap) perception following exposure to loud noise. A decreased ability to blunt the acoustic startle response (ASR) occurred under conditions prepulse inhibition (**A**), but was more pronounced during gap inhibition testing (**B,C**). Performance during ASR testing was recorded before noise exposure (Baseline), on the second day following noise exposure (Post Treatment 1) and on the third day following noise exposure (Post Treatment 2). (**A**) Relative to a full startle response (100%) animals were able to suppress the ASR by 30% during prepulse detection trials (baseline). (**B**) However, during post treatment 1 testing, at 12 kHz suppression was only 16% and by 48 hrs (post treatment 2) only 13%. (**C**) Also, at 16 kHz baseline gASR was 33% but by 24 hrs after noise exposure (post treatment 1) was only 21% of baseline and after 48 hrs (post treatment 2) 17% of baseline). Differing letters denote significance across time points, p≤0.05 was considered significant; error bars equal SEM.

Just as with the control and the salicylate groups, the baseline measures in the noise group indicated the ability of the animals to reliably inhibit pASR and gASR (Figure 5). Following exposure to a 10 kHz 118 dB 1/3 octave band sound animals sustained a substantive threshold shift (Table 1). Therefore the intensities for the pre-pulse (pASR) and background tones (gASR) were increased to levels that could be detected by the animals (70 and 95 dB). Despite the noise exposure, animals attenuated pASR responses across all frequencies at each time point tested (baseline – 30%, 24 hrs – 20%, 55 hrs – 24%) confirming that the animals were able to hear the pre-pulse (Figure 5A) with the decrease from 30% to 20 and 24% suggesting hearing loss effects. When tested for inhibition of gASR, there was a significant reduction in the ability to inhibit the acoustic startle reflex at both post-noise time points at the frequencies of 12 (baseline – 28%, 24 hrs – 16% p = 0.0079, 55 hrs – 14% p = .0008) and 16 kHz (baseline – 33%, 24 hrs – 21% p = 0.0079, 55 hrs – 17% p = 0.0003), reflecting the capacity of gASR to demonstrate not only the hearing loss but the perception of tinnitus as well. The results suggest that the tinnitus percepts were fairly tonal in nature and were experienced across two (12 kHz and 16 kHz) of the tested frequencies (Figure 5B–C).

**Figure 6:**
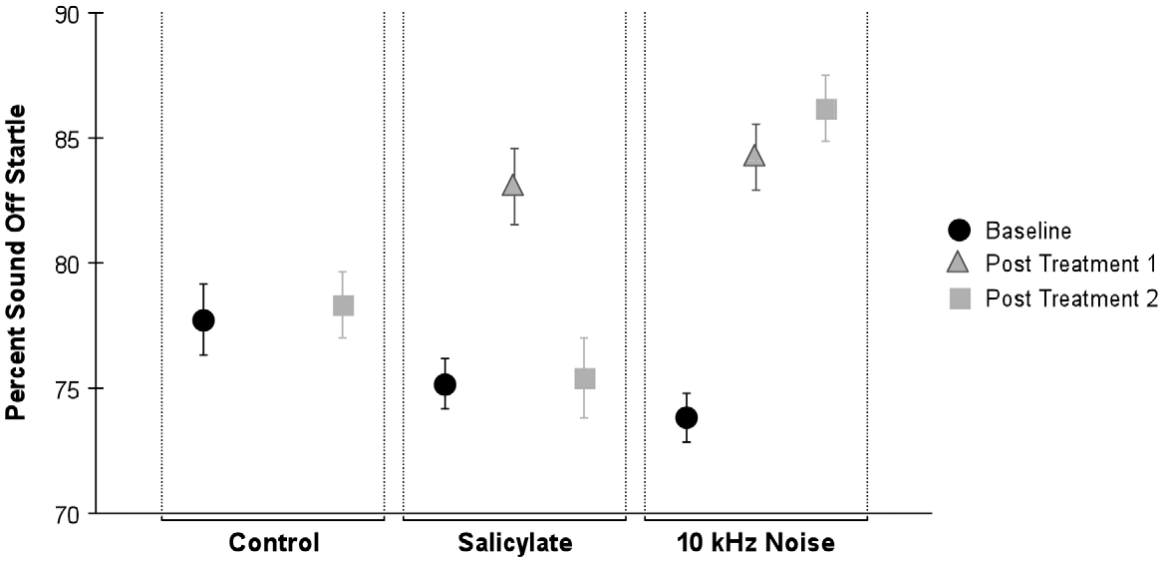
Summary graph of gap ASR comparing experimental groups (control, salicylate, noise) at three different time points (baseline, post treatment 1 and post treatment 2). For control animals the percent inhibition of startle remains constant. For the salicylate group post treatment 1 testing resulted in decreased inhibition of gASR, but by post treatment 2 testing, gASR had returned to normal levels. The 10 kHz noise group demonstrated a decreased ability to inhibit gASR at both time points following noise exposure when compared to baseline. Error bars equal SEM.

In animals that were not exposed to salicylate or noise, (the control group), the ability to inhibit the gASR remained constant (Figure 6). However, the salicylate group showed a decreased ability to inhibit gASR one hour after salicylate treatment, but by seven hours the ability to attenuate gASR had returned to normal levels. For noise-exposed animals, the ability to inhibit the gASR remained decreased at both time points following noise exposure (Figure 6). These results further support the idea that the tinnitus experienced by the salicylate group was transient while the noise-exposed group experienced a more uninterrupted tinnitus.

### MEMRI measures tinnitus-dependent differential regional brain activity

Salicylate and noise groups were given manganese 8 hours prior to imaging. This corresponded to a time when animals exhibited behavioral correlates of tinnitus (ASR testing of salicylate and noise groups).

All manganese-injected animals exhibited the expected high signal intensities in the anterior pituitary [31]. Raw signal intensities (before normalization to muscle) were analyzed at this location to verify that control and tinnitus groups experienced similar brain systemic manganese exposure: Between groups, raw signal intensities were similar at the anterior pituitary and adjacent muscle alike (one-way ANOVAs, P>0.05). These raw intensities were correlated (r = 0.49; P = 0.016), indicating that normalization to muscle would reduce inter-subject variability in brain signal intensities. After normalization to adjacent muscle, no group differences were found at the anterior pituitary (one-way ANOVA, P>0.05).

In non-auditory regions (listed in Table 3), no significant group differences in manganese uptake were found (one-way ANOVAs, P>0.05). For completeness, mean normalized signal intensities for these regions, along with specific two-tailed t-test comparisons of the control group to each experimental group, are presented in Table 3. Layer-specific analysis of the cortex revealed no group differences (P>0.05 for all cortical depths tested in all regions). Normalized signal intensities, averaged over cortical depths corresponding to layers II through VI, are similarly provided in Table 3.

**Table 3:** Effects of salicylate and noise exposure on neuronal activity in different brain regions as measured by manganese enhanced MRI.

Tinnitus Group		Control ^bcd^	Salicylate^ bcd^	Noise ^bcd^
Brain Region				
**Amyg** a	mean	160.8±4.0	158.7±6.8	169.7±6.6
	p-value		0.77	0.23
**Au1** a	mean	149.9±1.8	145.2±4.7	151.9±4.6
	p-value		0.39	0.69
AuD	mean	148.7±2.1	144.7±4.8	152.5±5.2
	p-value		0.46	0.52
**DCN** a	mean	210.3±6.8	242.4±12.1	209.9±5.2
	p-value		0.02	0.96
VCN	mean	189.0±5.6	189.5 ±3.9	187.4±9.0
	p-value		0.94	0.79
VCPO	mean	195.8±7.4	190.9±3.5	198.8±4.5
	p-value		0.55	0.73
**Floc** a	mean	172.0±5.2	168.3±1.9	169.1±3.4
	p-value		0.52	0.64
Pfloc	mean	162.7±4.1	158.0±2.1	158.0±3.1
	p-value		0.43	0.38
**LGN** a	mean	152.2±3.0	154.6±4.9	164.5±6.0
	p-value		0.94	0.79
MGN	mean	167.3±4.7	170.4±3.5	170.9±5.0
	p-value		0.59	0.59
**CNIC** a	mean	163.1±3.4	174.2±3.6	170.9±4.2
	p-value		0.03	0.15
DCIC	mean	176.7±3.2	195.2±5.0	186.4±2.2
	p-value		0.01	0.03
ECIC	mean	175.7±3.5	186.5±2.3	181.6±3.9
	p-value		0.02	0.25
SC	mean	162.9±4.7	169.8±4.4	169.8±4.8
	p-value		0.28	0.34
**TeA** a	mean	156.2±2.2	153.4±4.2	154.7±2.8
	p-value		0.58	0.69
V1	mean	148.2±2.5	147.0±3.7	154.5±4.3
	p-value		0.80	0.24
V1B	mean	150.0±3.4	156.0±3.3	157.3±2.7
	p-value		0.23	0.12
V1M	mean	147.2±3.4	154.8±3.8	157.9±3.1
	p-value		0.17	0.04
V2L	mean	154.9±2.7	155.8±3.1	153.5±3.2
	p-value		0.73	0.84
V2MM & V2ML	mean	145.9±2.6	149.0±4.3	154.0±4.1
	p-value		0.44	0.12

a Bold text indicates beginning of new brain region.

b Signal intensity, expressed as the percentage of adjacent muscle; arbitrary units (a.u.).

c Significance p≤0.05.

d Error (±) is expressed as SEM.

**Figure 7:**
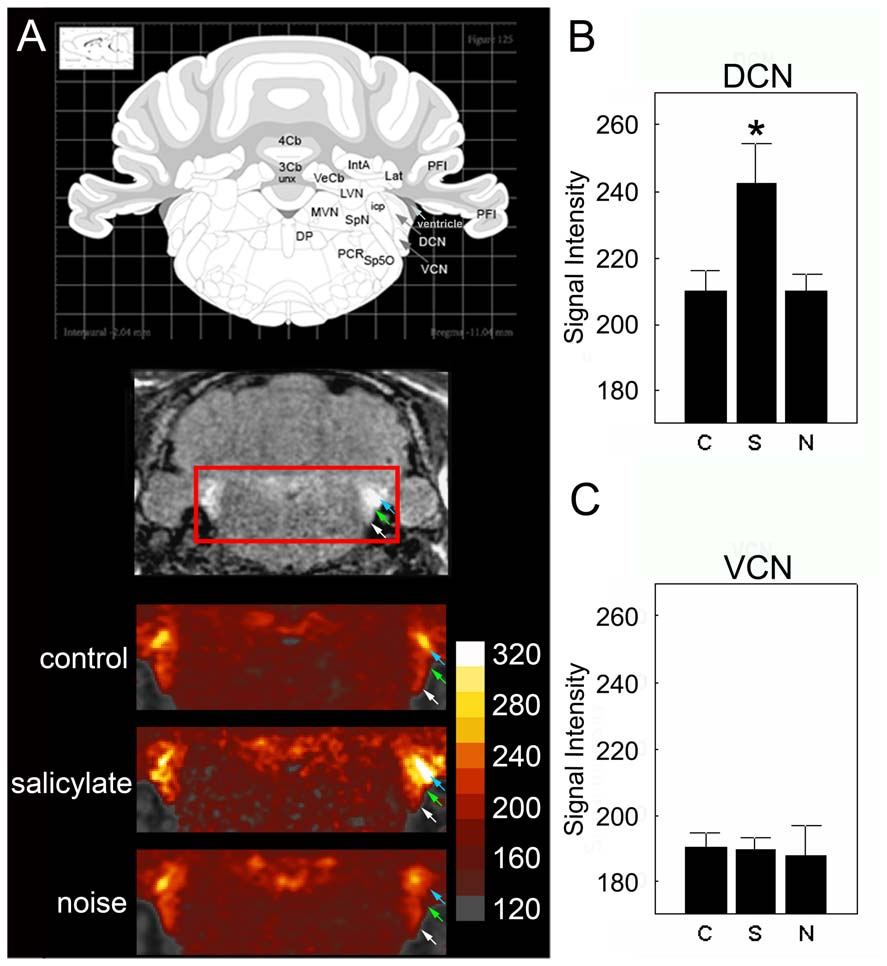
Salicyate increases MEMRI measured signal intensity in the DCN but not the VCN. In the dorsal cochlear nucleus (DCN) a significant increase in signal intensity was observed (**A–B**) in the salicylate treated group (242. 37±12.08; p = 0.02) when compared to controls (210.26±6.76), but no change was observed in the VCN (**A, C**). Noise exposure does not change MEMRI measured signal intensity in the DCN (209. 90±5.22; p = 0.02) or VCN (187. 41±9.02; p = 0.02) 48 hrs post noise exposure (A–C). Signal intensity is calculated as a percentage of nearby muscle. Asterisk denotes significance, p≤0.05; error bars equal StDev, C – control, S – salicylate, N - noise exposed In MRI panels white arrows indicate VCN, green arrows indicate DCN, and blue arrows indicate ventricular space.

**Figure 8:**
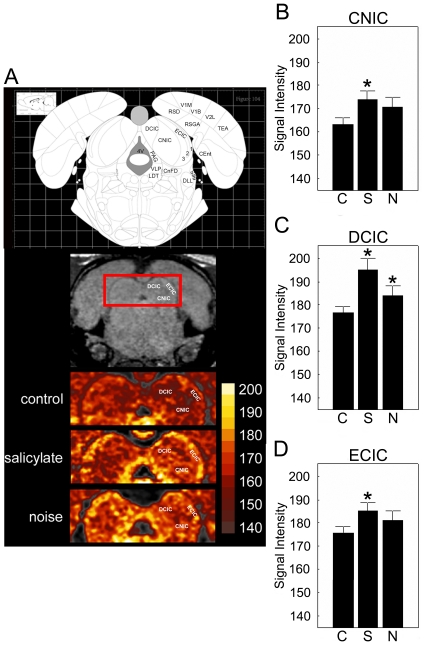
Effects of salicyate and noise exposure on MEMRI measured signal intensity in the inferior colliculus after three days of salicylate administration and 48 hrs post noise exposure. Signal intensity was compared across groups (A–D). In the salicylate group, manganese accumulation was supernormal in the central nucleus of the inferior colliculus (B; CNIC; 174. 20±3.60; p = 0.03), as well as the dorsal cortex (C; DCIC; 195. 21±4.96; p = 0.005) and external cortex (D; ECIC; 186. 52±2.35; p = 0.02) when compared to controls. In the noise exposed group, only the DCIC had increased signal intensity (186. 35±2.17; p = 0.03). Asterisks denote significance, p≤0.05; error bars equal StDev, C – control, S – salicylate, N - noise exposed.

Significant group differences were found in the DCN (Figure 7A–B; F_[2,17]_ = 5.22; P = 0.017) and the DCIC (Figure 8A–B; F_[2,17]_ = 7.60; P = 0.004). In post-hoc testing (two-tailed t-tests, uncorrected p-values reported), DCN manganese uptake was similar in noise-exposed and control groups (P>0.05), but elevated in the salicylate-exposed group (vs. noise P = 0.033; vs. control, P = 0.023; Figure 7A–B). In contrast, DCIC manganese uptake was elevated in animals with tinnitus (control vs. noise, P = 0.025; control vs. salicylate, P = 0.005); and there was no significant difference between salicylate- and noise-exposed groups (P>0.05; Figure 8A–B;). Statistical conclusions were identical when Tukey's Honestly Significant Difference test was used for post-hoc analysis (data not shown).

Besides the DCIC and DCN, no group differences in manganese uptake were found for any other sub-cortical structures (Figure 7–8 and Table 3). Sub-divisions of the inferior colliculus besides the DCIC (i.e. the ECIC and CNIC) failed to reach statistical significance (Figure 8), we found no evidence for group-wise regional differences within the inferior colliculus: Tests for both the ECIC and CNIC regions reached marginal significance (for each region, F_[2,17]_>2.73; P<0.10), and showed a similar between-group pattern of manganese uptake (Figure 8 i.e. control<salicylate; noise≈salicylate). In each group, normalized signal intensities trended lower in the ECIC and CNIC than in the DCIC (Table 3), though only for the CNIC was this difference significant in all three groups (paired two-tailed t-tests, P<0.002 for each). In control animals, VCN signal intensities were greater than in the DCIC, though still lower than in the DCN (paired two-tailed t-tests; p = 0.009 and 0.001 respectively, Table 3).

The pattern of regional signal intensities seen in the auditory regions of control animals (DCN>VCN>DCIC>CNIC>Aud) is intriguing. However, several factors unrelated to baseline neuronal activity may complicate between-region comparisons of MEMRI data (e.g. differences in myelination, proximity to ventricular space).

## Discussion

The literature suggests anomalous neural activity within central auditory pathways associated with tinnitus inducing drugs and noise [32], [33], [34], [35]. However, this is the first study in which all subjects undergoing tinnitus assessment also underwent imaging analysis in vivo. Using this approach we found that both salicylate and noise-induced tinnitus result in increased neuronal activity in DCIC neurons. The IC is the point of integration of all ascending and descending projections to the auditory cortex with the DCIC receiving the largest portion of the descending fibers [36], [37], [38], [39], [40], [41], [42]. The DCIC receives ascending projections from the DCN, the anteroventral cochlear nucleus (AVCN) and the dorsal nucleus of the lateral lemniscus (DNLL). Significant changes in activity were not observed in MGN or the auditory cortex. Future work may detect a spread of increased neuronal activity to those regions over time.

### ASR characterization of tinnitus percepts

First, we evaluated the tinnitus status of each rat. Using inhibition of startle techniques, pASR and gASR, we were able to correlate a decrease in the ability to inhibit the ASR at specific frequencies with the perception of tinnitus. These data support and extend previous results using this technique to characterize tinnitus.

#### ASR inhibition discriminates fluctuations in salicylate induced tinnitus

In the salicylate group, relative to a full startle response, all animals exhibited the ability to inhibit their ASR prior to treatment. One hour following salicylate administration, ASR suppression was diminished, though only for pre-startle tones centered on a frequency of 12 kHz (11–13 kHz). These results closely match previous studies, which identify salicylate-induced tinnitus with percepts ranging from ∼10–16 kHz [43], [44], [45], [46], [47], [48], [49]. Six hours after the last salicylate injection, gASR inhibition was not significantly different from baseline, likely reflecting the decrease in systemic salicylate and therefore decreased tinnitus perception. These results are consistent with those of other studies demonstrating that salicylate induced tinnitus in the rat is stable in terms of hearing loss and tinnitus perception by the third day of treatment and that the perception of tinnitus is reversible [43], [44], [45], [46], [47], [48], [49].

#### ASR inhibition suggests salicylate induced tinnitus results in hyperacusis

We also tested the performance of the animals in pASR testing. We typically use pASR to verify that animals can detect tones and thus negate hearing loss as a factor on performance. Prior to treatment animals were able to inhibit their ASR in response to a pre-pulse. Following salicylate treatment the inhibition was more robust. Previous studies as well as the current study have noted a general increase in startle amplitude for animals administered salicylate [49], [50], [51]. Because each startle event is compared to the full startle response from each session, the ability to suppress the startle response following salicylate administration would be expected to diminish. This was not the case in the present work, suggesting an improvement in auditory perception leading to a heightened suppression of the startle response that has been termed “hyperacusis”. This type of sensitivity to loud noise is often observed in human subjects with tinnitus.

#### ASR inhibition differentiates frequency specific changes in noise-induced tinnitus

In animal models, noise has also been used to induce tinnitus, as defined via behavioral testing, [17], [52], [53]. In addition to the current study, other reports demonstrate that exposure to the equivalent of a 10 kHz 118 dB 1/3 octave band tone results in tinnitus, but also profound deafness [54]. Since the ASR performance is dependent upon hearing, in the current study one ear in each animal from the noise group was plugged during the noise exposure, leaving that ear capable of detecting the tones for pASR and gASR in addition to resulting in an asymmetric hearing loss. The fact that only one functional ear is necessary for pASR and gASR tinnitus assessment has previously been reported [17]. To compensate for increased hearing thresholds in the plugged ear, intensity levels were raised for ASR testing. To determine whether the animals had the ability to hear well enough to inhibit the ASR, we first tested pASR. Animals were able to consistently decrease their startle amplitude from the full startle response (i.e. 100%). Next, performance on the gASR test was assessed and both 12 kHz and 16 kHz showed evidence of tinnitus-like percepts at both time points (24 and 48) hours following noise exposure.

Thus, a total of two ASR sessions were recorded following treatment with either salicylate or noise. The second post treatment ASR test was given 54 to 56 hours following the initial induction of tinnitus. For the salicylate group this time point corresponded with seven hours following the day three salicylate injection (post treatment 2 ASR) and results from gASR testing showed no significant difference between the post treatment 2 gASR (7 hours following the day three salicylate injection) and baseline measures perhaps indicating a lack or decrease of tinnitus percepts. These results support previous findings showing the diminution of tinnitus percepts within four–six hours following salicylate administration [51]. For the noise group, frequencies associated with tinnitus percepts post-treatment, continued to exhibit gASR measures significantly different from baseline measures even on the second day of assessment following exposure. Our results confirm findings from previous studies showing that gASR is an advantageous and reliable technique for assessment of tinnitus whether induced by salicylate [30] or noise [17], [51]. Given our ASR results in both the salicylate and noise groups we concluded that the animals experienced tinnitus-like percepts. However, ASR tests do not provide insight into specific tinnitus-related brain pathophysiology such as altered regulation of ions (i.e., calcium).

### MEMRI demonstrates differential brain region activation in salicylate and noise induced tinnitus

Manganese, an activity-dependent contrast agent, accumulates in active neurons through, for example, voltage-gated calcium channels. Increased neuronal activity results in increased cellular manganese uptake. For a given region of interest then, differences in manganese accumulation, measured non-invasively with MRI, reflect differences in neuronal activity during the period of manganese accumulation, before anesthesia and MR imaging [55]. The use of MEMRI avoids a major drawback of doing functional studies in MRI scanners for testing of central auditory structures: an extremely noisy scanner environment. Since manganese uptake is activity dependent and loss from the cell is slow, neuronal activity in awake animals can thus be encoded outside of the magnet with activity readout hours later.

In the present study, MRI images with high spatial resolution were needed to analyze the relatively small regions in the auditory brainstem. To this end, brain manganese levels were measured using signal intensities from T_1_-weighted images. Normalization to adjacent tissue is a common part of this approach, either to a negative-control brain region [56], [57] or to nearby muscle [18], [27], [55], [58]. The tissue used for normalization must be carefully selected, as underlying group differences there may obscure or over-emphasize group differences in regions of experimental interest. The quality of normalization can be gauged by evaluating negative-control regions, where no group differences are expected. In the present work, negative findings at the anterior pituitary and throughout the visual system – covering a broad range of enhancement levels – are consistent with appropriate normalization, as are the heterogeneous results for neighboring and identically-normalized auditory regions (e.g. VCN vs. DCN). Future work may benefit from T_1_ mapping of the brain [31] since this will be sensitive to a broader range of tissue manganese concentrations while being unaffected by receive-coil artifacts – the primary motive for normalizing signal intensities in the present study.

Recent studies have suggested that MEMRI can be used to image the auditory brainstem [18], [20], [59]. For example, MEMRI was used to identify tinnitus related activated brain regions ex vivo [18]. Previous lower-resolution studies have utilized PET and MRI to identify tinnitus associated brain regions that show changes in neuronal activity [60], however the low resolution of these techniques has prevented detailed identification of brain regions involved. Further corroboration of the sensitivity of the MEMRI for use in the auditory system is the interesting correlation of signal intensity and brain regions in normal hearing controls. We found higher signal intensities in the DCN when compared to the VCN. This fits well with other studies in which the VCN is reported to have lower levels of spontaneous neuronal activity as assessed via electrophysiology when compared to the DCN [61], [62], [63]. Following deafferentation, the DCN continues to show spontaneous activity while the VCN is quieted [64], [65], [66]. Our results support these findings with the DCN exhibiting significantly more manganese uptake than the VCN in normal hearing animals and with only DCN showing changes in activity following tinnitus induction.

#### MEMRI associated changes in neuronal activity within auditory pathways occurred bilaterally

In the current study, brains of animals with salicylate and noise induced tinnitus-like percepts were compared during the early stages of tinnitus onset. In the DCN and DCIC, there were no differences observed in manganese uptake in the right and left sides. Since the injections of salicylate were systemic, this result was not surprising. However, in previous studies where a tone was presented directly into a single ear, the changes in neuronal activity were lateralized, [5], [18], [67] but when the IC was studied there was no difference in hemispheres (Melcher 2009). In the current study, a plug was inserted into one ear for protection against profound hearing loss. This “protection” resulted in an asymmetry in terms of hearing loss in the right versus left ears possibly contributing to lateralized tinnitus percepts. However our imaging studies suggest that the tinnitus that was induced, at least during the early stages we tested, was not hemisphere specific. We surmise that while the plug was sufficient to preserve hearing well enough for animals to successfully perform during ASR testing, the plug did not prevent tinnitus-associated uptake of manganese in central pathways associated with the plugged ear.

#### Specific tinnitus induction results in differential manganese uptake

In the cochlear nucleus only the DCN showed an increase in manganese uptake in our study, a result only observed in the salicylate group. Salicylate has been shown to increase spontaneous activity in the DCN using extracellular recording and with imaging techniques. Using electrophysiology and a very similar noise exposure to that of our study, another study has previously shown increases in neuronal spontaneous activity in the DCN [8]. In the current study we did not observe changes in the DCN of animals from the noise group. Perhaps the observed differences can be attributed to the time point we used for assessment. In that electrophysiology study DCN activity was initially decreased following exposure with increases in activity observed by five days. We assessed changes in neuronal activity in the noise group two days following the noise exposure. Assessing neuronal activity at a later time point, when increased DCN activity is expected from noise-induced tinnitus may yield significant differences from baseline. In a previous tinnitus related MEMRI study, while no changes in neuronal activity were observed in the DCN following noise there were increases in manganese uptake in the PVCN [18]. This discrepancy between that study and the current one may be due to the difference in noise exposure. The previous study used a noise exposure that resulted in a temporary threshold shift that had returned to normal levels prior to manganese injection, while the acoustic exposure in the current study results in a permanent threshold shift and profound deafness. In the previous MEMRI study, manganese was administered to animals showing psychophysical evidence of tinnitus months after the sound exposure. Therefore results from that study would include changes in neuronal activity reflecting uptake due to chronic tinnitus and not the early stages of tinnitus reported in the current study. More work is now needed to resolve these issues.

In the inferior colliculus, both salicylate- and noise-induced tinnitus resulted in significantly elevations in manganese uptake. These results suggest an important role of IC, specifically DCIC, involvement in tinnitus-related neuronal activity. Consistent with this, tinnitus-associated changes in neuronal activity within the IC have been reported following salicylate [60], [67], [68], [69] and noise alike [18], [53], [70], [71], [72].

In the present study, we found no significant activity differences in the auditory cortex. This coincides with recent studies in the rat using electrophysiology where changes in spike amplitude, but not spontaneous activity were observed following salicylate and noise [72]. However, in contrast to our results, PET studies in humans and animals as well as autoradiography have suggested that changes in auditory cortex activity occur in subjects with tinnitus. One possible reason for this differential finding may be use of guinea pigs in these earlier studies instead of rats, and/or use of a different tracer (deoxyglucose), rather than manganese. Although our results are based on linearization of the auditory cortices, which provides a sensitive assessment of changes in individual cortical layers [55], we did not find significant differences in manganese uptake in the auditory cortex.

### Conclusion

Our goal was to study in awake rats changes in neuronal activity in tinnitus related brain regions that are common to different types of tinnitus using two complementary approaches (ASR and MEMRI). Since the current study focused on similarities and differences emerging hours – days following tinnitus onset and the mechanisms involving the maintenance of chronic may be very different, in the future this approach can be expanded for use in longitudinal studies and in evaluation of treatment efficacy regardless of time following onset or method of induction. The combination of ASR and MEMRI should prove to be quite a powerful combination since MEMRI has high spatial resolution, is not susceptible to scanner noise artifacts and both manganese uptake and ASR can be measured repeatedly.

## Methods

### Ethics Statement

All procedures were approved by the Wayne State School of Medicine “Laboratory Animal Care and Use Committee” (PHS Animal Welfare Assurance number A3310-01) and conform to the National Institutes of Health guidelines.

### Subjects

Twenty-four male Sprague Dawley rats were obtained from Charles River Laboratory. All rats were 2–3 months old weighing 250 g–270 g. The rats were individually housed and maintained at 25°C with a 12 hour light dark cycle. Three groups of animals were studied. The control group consisted of animals in which tinnitus was not induced (n = 8) while in the other two groups tinnitus was induced with either salicylate (n = 6) or noise (n = 6). Limited testing was also performed in animals imaged without manganese (n = 2), animals tested with ASR without Mn^2+^, but not imaged (n = 2), animals tested with ABR after manganese (n = 2), and ASR 4–10 days after noise exposure (n = 2).

### Auditory Brainstem Response (ABR) Testing

To determine the hearing thresholds for each group, ABR measures were recorded before and after treatment, and after MnCl_2_ administration. Animals were anesthetized with i.m. injections of ketamine HCL (50 mg/kg) and xylazine (2 mg/kg) and placed in a sound attenuating booth (Kinder Scientific Poway, CA). Sub-dermal electrodes were inserted below the pinna (reference electrode), on the top of the head (active electrode), and below the contralateral pinna (ground electrode). A Beyer speaker (Beyer, Germany) was placed into the external ear canal and thresholds were obtained from tone bursts 5 ms in duration for 340 trials for each of three frequencies (4, 12 and 20 kHz) up to 100 dB. Sound was generated and evoked potentials were recorded amplified and filtered using Daqarta (Data AcQuisition And Real-Time Analysis) digital signal processing software package (v4.51, Interstellar Research, Michigan) and a Grass preamplifier. Hearing thresholds were established by determining the lowest intensity of a tone between 0–100 dB that elicits a response at each frequency.

### Control Protocol

Testing of the animals in the control group were interspersed with testing of animals in the tinnitus groups. Each of the animals had 2–3 days of baseline ASR's recorded. Eight hours prior to imaging, most control animals (N = 8) were administered 66 mg MnCl_2_·H_2_0/kg (i.p.). This manganese dose and timing have been shown adequate for functional brain imaging [55]. Two hours prior to imaging, a post-MnCl_2_ ASR was recorded (Figure 2A). In addition, two of the control rats were imaged without MnCl_2_ to verify that appropriate enhancement took place in all regions of interest.

### Salicylate Protocol

Baseline ASR tests were recorded in each animal (N = 6) for two to three days. Next, animals received a daily i.p. injection (300 mg/kg) of sodium salicylate (Sigma Aldrich) in physiological saline for three days. One hour following the second day of salicylate administration a post treatment-ASR was recorded followed by ABR testing for hearing thresholds. MnCl_2_ (Sigma, Aldrich) was administered (i.p.; 66 mg MnCl_2_·H_2_O/kg) one hour after the third salicylate injection, eight hours prior to imaging. A post MnCl_2_ ASR was recorded two hours prior to imaging (Figure 2B).

### Noise Exposure Protocol

Baseline ASR tests were recorded in each animal (N = 6) for two to three days. For acoustic exposure, animals were first placed into individual cages that were set on racks in a custom built noise exposure booth. We have previously shown that the noise exposure used here results in a permanent shift in hearing threshold [54]. Since hearing is necessary for ASR testing, noise exposure was attenuated for one ear: 30 minutes prior to noise exposure, an ear plug was inserted into either the right or the left ear canal (unilaterally, semi-randomly), and the auricle of the plugged ear was folded and secured with a single stitch. Gauze was taped over the stitched ear and a paper neck collar applied during the noise exposure to prevent the animal from injuring the ear. Noise was generated using DaqGen software (Daqarta, Interstellar Research) channeled through 16 overhead speakers and Grass preamplifiers controlled using an Apple computer. Animals were exposed for 4 hours to a 10 kHz, 118 dB one-third octave band tone. To determine the effects of noise exposure on both the plugged and unplugged ears, ABRs and ASRs (using levels that were detectable by the noise exposed animals – 75 and 90 dB) were recorded 24 hrs following sound exposure. On the second day post sound exposure, MnCl_2_ (66 mg MnCl_2_·H_2_O/kg) was administered (i.p.) and imaging was conducted eight hours later, with a post MnCl_2_ ASR recorded two hours prior to imaging (Figure 2C).

### Acoustic Startle Reflex (ASR) Testing

Based on previous studies, the acoustic startle reflex provides a useful and reliable method for detecting salicylate and noise induced tinnitus in rats [17], [49]. All tests were performed in a noise attenuation chamber with customized hardware and software (Kinder Scientific, Poway, CA). Inside the chamber, animals were placed inside a plexi-glass box resting on a piezoelectric force transducer in a calibrated sound field. The adjustable ceiling of the box was positioned above the animal to constrict excessive movement. All ASR tests were conducted in the light. The ceiling of the box has slits and sits 15 cm from the speaker located at the top of the chamber. The output of the piezo transducer was amplified, filtered, and the root mean square of the waveform measured to estimate startle amplitude.

For each animal both pre-pulse inhibition (pASR) and gap detection inhibition (gASR) of acoustic startle was conducted. First pASR testing was performed, immediately followed by gASR testing. Testing resulted in animals spending no more than one hour per day in the testing chamber. pASR testing aids in the interpretation of gASR data, providing a control for both hearing loss and temporal processing dysfunction. In our paradigm, deficits in gASR accompanied by normal pASR represent the presence of tinnitus. For the pASR testing, pre-startle tones were presented at 4,8,12, 16, 20, and 24 kHz at levels of 45 and 60 dB in all groups with the exception of the Noise Group: Since those animals experienced hearing loss, levels of 75 and 90 dB were used in ASR conducted post noise exposure. Each pre-startle tone was presented for 20 ms, in a silent background 50 ms before the sound off startle (120 dB broad band noise for 50 ms). For each ASR test (pre-pulse - pASR or gap - gASR) a total of 10 trials were run for each frequency at each intensity as well as in the sound off startle condition for pASR and in both the sound off startle condition and the sound in startle condition for gASR. The trials were presented in a pseudo-randomized order, with inter-trial intervals randomized between 1 and 6 s.

During gASR testing, a background tone of either, 4, 8, 12, 16, 20, or 24 kHz at 45 and 60 dB, (or 75 and 90 dB in post noise exposed animals) was presented. A silent gap lasting 20 ms was embedded in the tone 50 ms prior to the startle (120 dB wide band noise). To determine a maximum startle response, a startle was also elicited without any background tone (sound off startle). For each frequency at each sound level, with 10 trials recorded for each condition as with pASR.

### MEMRI Image Acquisition

Eight hours post MnCl_2_ injection, animals were anesthetized with urethane 3.9 ml/kg i.p. (36% solution, prepared fresh daily; Aldrich) and promptly scanned. The timing between injection and scanning was based on previous work showing that brain manganese accumulation is adequate for functional imaging after eight hours [55], as well as a study outlining a detailed time-course for manganese enhancement in the mouse brain. In this approach, MEMRI data report on brain activity between injection and scanning – while animals are awake and freely-moving – with anesthetic choice or scanning environment having little opportunity to influence results [31]. To specifically check the effects of urethane on manganese uptake, we have performed retinal functional studies with MEMRI in the same animal before and after death. We find no differences in retinal signal intensity (data not shown). Thus, there is little reason to think that urethane influences the manganese signal.

For the duration of the scan, each animal was placed on a heated re-circulating water pad with a rectal thermometer to monitor and maintain core body temperature. Scans were performed on a 4.7 T Bruker Avance System using a whole body transmit-only coil and a 3 cm internal diameter receive-only coil. Individual images were acquired using a 3D RARE sequence (repetition time [TR] 330 ms; echo time [TE] 6.9 ms, with a RARE factor of 8, for an effective echo time of 28 ms; number of acquisitions [NA] 4; matrix size 216×248×120; field of view [FOV] 2.81×3.22×3.12 cm^3^; providing a resolution of 130 µm×130 µm×260 µm; 37 min/image). All animals were alive when scanning was complete. As described previously [55], we also scanned a “phantom” roughly the size of a rat's head (containing a 10∶1 mixture of water and 0.67 mM MnCl_2_ in saline) using the same parameters employed in animals for later modeling of signal intensity changes as a function of distance from the surface coil.

### ASR Data Analysis

In the pASR paradigm results were calculated as the force of the startle response following the pre-pulse preceded startle noise, divided by the sound off (no background sound) startle multiplied by 100. The results are therefore reported as the percent inhibition of the sound off startle. For the gASR paradigm the data were calculated in the same way except for each trial at each frequency and sound level the maximum startle response was recorded both in the presence of the background tone and in a silent background. Data were analyzed using StatView for between group comparisons and SPSS software v 7.0 for repeated measures analysis of variance (for within group comparisons) to determine significant differences and post hoc comparisons to determine which means contributed to the observed effects.

### MEMRI Image Analysis

For these experiments increased manganese uptake was equated to changes in ion regulation and increased neuronal activity. Therefore the terms are used interchangeably throughout the text. Image analysis proceeded, with slight modification, as detailed elsewhere [55]. Briefly, the influence of surface coil position on signal intensity was modeled, then corrected for within each image using R scripts developed in-house (v.2.9.0; R Development Core Team (2009); http://www.R-project.org). Next, average signal intensity was measured from several manually-defined regions of interest (ROIs) using MRIcro v.1.40, [73] with tracings of each structure guided by the Paxinos and Watson (6^th^ Edition; 2007) rat brain atlas including several sub-cortical auditory and non-auditory regions: the medial geniculate nucleus (MGN), the central nucleus, dorsal cortex, and external cortex of the inferior colliculus (CNIC, DCIC and ECIC, respectively), the dorsal cochlear nucleus (DCN) and ventral cochlear nucleus (VCN), the paraflocculus (Pfloc), flocculus (Floc), amygdala (amyg), superior colliculus (sup coll), and lateral geniculate nucleus (LGN). Spherical ROIs were used to characterize the CNIC, MGN, LGN, and sup coll (radius: 520 µm). Hand-drawn ROIs spanning three consecutive coronal slices (each 260 µm thick) were used in all other cases, except for the DCN: Owing to the small size of the DCN, its ROI was drawn in two consecutive slices with the broadest cross-sectional areas. Each anatomical region was identified both by its broad contours, and proximity to prominent adjacent structures: The contours of the paraflocculus and cochlear nuclei, for instance, are clearly visible in several coronal slices (e.g. Figure 7A), simplifying comparisons to the Paxinos and Watson atlas and facilitating fine localization of the DCN and VCN. Hand-drawn ROIs were placed at the rostrocaudal center of each anatomical region, and drawn to occupy the entire coronal profile of each region, excluding a buffer (≥1 voxel wide, depending on the ROI) at borders with neighboring brain regions and tissues (e.g. choroid plexus). Appropriate ROI placement was confirmed in parasagittal views, and the subject-to-subject consistency of ROI placement was checked by at least two of the authors.

Several cortical regions, both traditionally auditory – primary auditory cortex (Au1) and dorsal zone of auditory cortex (AuD), and regions not traditionally associated with auditory pathways - temporal association area (TeA), lateral (V2L) and medial (V2M) secondary visual cortex, and the monocular (V1M), binocular (V1B), and rostral (V1) portions of the primary visual cortex – were analyzed in a manner which preserves cortical layer-specific information as previously described [55]. Briefly, high-order polynomials were fit to the brain/no-brain border in coronal slices. Lines perpendicular to the polynomials were automatically drawn, and tilted in the rostrocaudal plane to be consistent with the brain surface. Brain signal intensities were evenly sampled along these lines and organized as a linearized image of the cortex. Here, a linearized version of the Paxinos and Watson atlas (6^th^ Edition; 2007) was used to divide the linearized cortex into each ROI. Average profiles of signal intensity as a function of cortical depth were then generated for each ROI, and data at cortical depths corresponding to layers II through IV used for between-subject comparisons.

For all functional comparisons of MEMRI data, brain signal intensities were normalized to adjacent tissue, similar to the approach used elsewhere [27], [55], [56]. Here, ROI signal intensities (SI) were normalized adjacent muscle (normalized ROI SI = ROI SI/muscle SI * 100). Muscle normalization can compensate for any signal intensity gradients that remain after image processing, as well as potential inter-individual differences in peripheral MnCl_2_ uptake (e.g. liver sequestration). Normalization to a non-auditory brain region, which might better-provide those benefits, was also considered. However, association with tinnitus for most brain regions remains unclear.

To check the quality of muscle normalization, additional analyses were carried out at the anterior pituitary and adjacent muscle. Because the anterior pituitary sits outside of the blood-brain-barrier, it has been used in previous work to gauge non-specific brain enhancement due to systemic increases in manganese [74]. Here, anterior pituitary SI was analyzed using two spherical ROIs (radius: 390 µm) placed to the left and right of midline in coronal sections. To gauge whether muscle normalization would reduce inter-individual variability in brain data, we tested for a positive correlation between pre-normalization SIs for the pituitary and the adjacent muscle (linear regression). To test whether control and tinnitus groups experienced similar levels of manganese uptake, we use one-way ANOVAs to compare pre-normalization SIs for the pituitary and the adjacent muscle alike, as well as normalized pituitary SIs.

Functional comparisons of MEMRI data from controls to each tinnitus group were carried out by performing one-way ANOVAs at each brain region. Significant ANOVA results were further tested using post-hoc t-tests.
